# World Class Parasites: Vol. X, Schistosomiasis

**DOI:** 10.3201/eid1203.051537

**Published:** 2006-03

**Authors:** James McKerrow

**Affiliations:** * University of California at San Francisco, San Francisco, California, USA

**Keywords:** book review, world class parasistes book, W. Evan Secor, Daniel G. Colley

This compendium of reviews on the human parasitic infection, schistosomiasis, is a timely and much-needed resource for both research investigators and clinical personnel in many scientific and medical fields. Schistosomiasis is a major global health problem that affects hundreds of millions of people. It has been called one of the "great neglected diseases" because of the relative lack of interest by the pharmaceutical industry in developing diagnostics, drugs, and vaccines for it. The parasites that cause this disease, several related species of blood flukes, are also fascinating subjects for biologic research because of their complexity, relatively long lifespan, and remarkable host-parasite biology. To paraphrase Daniel Colley, one of the editors: "Analysis of this infection has contributed as much to our understanding of the human immune response and host parasite interactions as science has contributed to the control of the disease."

What sets this book apart is its readability for audiences from diverse backgrounds. This is in large part due to the thoughtfulness the editors put into choosing authors and topics, and perhaps most importantly, the relative brevity of each chapter. This latter point cannot be overemphasized since many compendiums overwhelm the reader with lengthy and dense material. This book is, therefore, especially valuable as a resource for students, postdoctoral fellows, and first-time investigators in this field.

One obvious conclusion that a reader new to the field will take away from this book is the diversity of research in this field. This reflects, as the editors note, a newfound intellectual energy born of the successful (albeit fledgling) applications of molecular genetic techniques to the study of the host-parasite relationship in schistosomiasis. At the same time, a shortcoming of the book is the relative lack of attention to the biochemistry of schistosomes, which is a key foundation for understanding how the parasite ticks and identifying new targets for chemotherapy. The work of investigators like John Dalton and Conor Caffrey on schistosome digestion of host proteins, for example, gets bare mention. This may be an inevitable consequence of the authors coming from a background of schistosome immunology, which is well represented in the book.

Another shortcoming is probably inescapable. As a field like schistosomiasis research enters a period of rapid and exciting advances, it is difficult for a book to keep up with the most recently published work. For example, many insights into the host-parasite relationship are becoming clear from proteomics analysis by groups like that of Alan Wilson at the University of York. Perhaps this criticism is actually reflecting good news as the field is reenergized.

Colley correctly points out that the key to the future of research on this disease, and new approaches to its control, lies in recruiting a new generation of scientists and clinicians with an interest in the disease. I have been struck by the number of graduate students and MD/PhD students who have expressed a renewed interest in schistosome research over the past year. Like Colley, I believe this reflects the exciting technologic advances and novel approaches to understand the biology of this fascinating organism that are beginning to show success. If we are right that a new generation of young scientists are testing the waters of schistosome research, this excellent compendium could not come at a better time.

